# Machine learning identifies ferroptosis-related genes as potential diagnostic biomarkers for osteoarthritis

**DOI:** 10.3389/fendo.2023.1198763

**Published:** 2023-06-12

**Authors:** Yue Qiu, Jun Yao, Lin Li, Meimei Xiao, Jinzhi Meng, Xing Huang, Yang Cai, Zhenpei Wen, Junpu Huang, Miaomiao Zhu, Siyuan Chen, Xingqing Long, Jingqi Li

**Affiliations:** ^1^ Bone and Joint Surgery, The First Affiliated Hospital of Guangxi Medical University, Nanning, China; ^2^ First Clinical Medical College, Guangxi Medical University, Nanning, China

**Keywords:** osteoarthritis, ferroptosis, immune infiltration, targeted therapy, machine learning

## Abstract

**Background:**

Osteoarthritis (OA) is one of the most common forms of degenerative arthritis and a major cause of pain and disability. Ferroptosis, a novel mode of cell death, has been verified to participate in the development of OA, but its mechanism is still unclear. This paper analyzed the ferroptosis-related genes (FRGs) in OA and explored their potential clinical value.

**Methods:**

We downloaded data through the GEO database and screened for DEGs. Subsequently, FRGs were obtained using two machine learning methods, LASSO regression and SVM-RFE. The accuracy of the FRGs as disease diagnosis was identified using ROC curves and externally validated. The CIBERSORT analyzed the immune microenvironment rug regulatory network constructed through the DGIdb. The competitive endogenous RNA (ceRNA) visualization network was constructed to search for possible therapeutic targets. The expression levels of FRGs were verified by qRT-PCR and immunohistochemistry.

**Results:**

In this study, we found 4 FRGs. The ROC curve showed that the combined 4 FRGs had the highest diagnostic value. Functional enrichment analysis showed that the 4 FRGs in OA could influence the development of OA through biological oxidative stress, immune response, and other processes. qRT-PCR and immunohistochemistry verified the expression of these key genes, further confirming our findings. Monocytes and macrophages are heavily infiltrated in OA tissues, and the persistent state of immune activation may promote the progression of OA. ETHINYL ESTRADIOL was a possible targeted therapeutic agent for OA. Meanwhile, ceRNA network analysis identified some lncRNAs that could regulate the FRGs.

**Conclusion:**

We identify 4 FRGs (AQP8, BRD7, IFNA4, and ARHGEF26-AS1) closely associated with bio-oxidative stress and immune response, which may become early diagnostic and therapeutic targets for OA.

## 1 Introduction

As the most prevalent degenerative arthritis, OA affects multiple joints such as knees, lumbar, hips, facet, and temporomandibular and becomes irreversible when it develops to a certain extent (1). Currently, non-pharmacologic and pharmacologic therapies are employed to treat OA. Among the pharmacological treatments, analgesics and NSAIDs are the current options for treating OA, and their effectiveness has been proven (2). However, these drugs can be used as symptomatic treatment but cannot change the nature of the disease. Therefore, it is necessary to explore the occurrence and progression mechanism of OA and identify targeted therapeutic targets that are beneficial to the early prevention and treatment of OA.

OA is a complex pathological process, and one of its most prominent features is the degradation of articular cartilage (3). As chondrocytes are the only biological constituents of articular cartilage, researchers have revealed that the occurrence of ferroptosis in these cells is closely associated with the progression of OA (4). As a new type of programmed cell death, the conception of “ferroptosis” was initially given by Stockwell et al. in 2012 and has been extensively researched in recent years (5). Excessive production and accumulation of lipid peroxides and reactive oxygen species (ROS) are the typical characteristics of ferroptosis, and its essence is the unbalance between the generation and degradation of intracellular lipid ROS (6). When cellular cysteine (e.g., Erastin) transport proteins are inhibited, intracellular glutathione (GSH) is depleted, eventually resulting in the inactivation of glutathione peroxidase (GPX4) and the accumulation of lipid peroxidation, which can induce cellular ferroptosis after reaching a certain level. Inhibiting GPX4 enzymes (e.g., RSL3) can also directly accelerate this effect, and iron ion chelators can inhibit this process (7). These findings suggest that inhibition of ferroptosis is a new direction to prevent the progression of OA, and exploring the immune infiltration in OA and its relationship with ferroptosis will help analyze the pathogenesis of ferroptosis in OA.

Accumulation of lipid peroxidation is a dangerous factor for the progression of OA, and dysregulation of iron homeostasis mediated by pro-inflammatory cytokines plays a critical part in iron overload-induced OA progression (4, 8). Pro-inflammatory cytokines, including interleukin-1β (IL-1β) and tumor necrosis factor-a (INF-a), are the main components of inflammation and contribute to cartilage damage in OA. Ding et al. revealed that IL-1β or INF-a could lead to iron overload by increasing iron influx and decreasing iron outflow (9). The PI3K/AKT/CONFAB pathway associated with IL-1β and mediated by enzyme-linked receptors can control cellular life processes. It can be activated with the assistance of various growth factors, cytokines, extracellular matrix, etc., and affect the NF-κB pathway closely associated with inflammatory mediators in tissue inflammation, tumor development and invasion, cell proliferation and apoptosis. Li et al. reported that melatonin could prevent steroid-induced osteoporosis by preventing ferroptosis via triggering the PI3K/AKT/mTOR signaling pathway (10). Chen et al. proposed that curcumin triggers ferroptosis by inhibiting the PI3K/Akt/mTOR signaling pathway, thereby inhibiting the proliferation of colorectal cancer cells (11). So far, ferroptosis-related pathways have been related to many degenerative diseases, carcinogenesis, ischemia-reperfusion injury, and stroke (11). However, the mechanism of ferroptosis in OA is still unclear, and as far as we know, there are still no relevant studies exploring FRGs and their regulatory mechanisms in OA progression.

From a comprehensive perspective, exploring ferroptosis’s clinical significance and underlying molecular mechanisms in OA is important. This study collected samples from OA patients and healthy individuals from the GEO database. Subsequently, we used bioinformatics methods to screen OA differentially expressed genes (DEGs) and feature genes linked to ferroptosis and carried out immune infiltration analysis to analyze their correlations, providing new strategies and directions for treating OA. Finally, we found 149 DEGs, with 4 feature genes identified as potential biomarkers of ferroptosis in OA.

## 2 Materials and methods

### 2.1 Data acquisition and processing

The Gene Expression Omnibus (GEO) database was used to search and download qualified expression datasets. The downloaded data sets GSE82107, GSE10575, GSE19060, GSE16464, and GSE27390 were used in this study. The probe matrix was transformed into the gene expression matrix through the annotation information of the platform files. The list file of FRGs was downloaded from the FerrDb database (http://www.zhounan.org/ferrdb/current/). The expression matrix of FRGs was extracted using the “limma” package in R.

### 2.2 Data quality control and date analysis

We use the “limma” package of R software to standardize the data of the above data sets, and take log2 to process the data with great differences, and finally merge them. Using R software, the combined data set is analyzed by relative logarithmic expression (RLE), and the related box diagram is made (**Supplementary Material 1**). The “pheatmap” package was performed to draw the heatmap of DEGs, and the difference between FRGs and their expression between the experimental and control groups was screened. The correlation heatmap was created using the “corrplot” package. The “clusterProfiler” package in R was used to perform the Gene Ontology (GO) analysis and Kyoto Encyclopedia of Genes and Genomes (KEGG) analysis with the threshold as *p*-value <0.05.

### 2.3 Screening feature genes

Two machine-learning algorithms were used to predict disease status to identify significant feature genes. LASSO is a regression analysis algorithm that improves prediction accuracy through regularization. As a popular supervised machine-learning technique, the Support vector machine (SVM) is frequently applied for classification and regression. To avoid overfitting, the optimal genes were selected from the meta-data cohort using an RFE algorithm (12). The LASSO regression and SVM-RFE were applied to screen the feature genes. The genes obtained by the two methods were taken to be intersected; the overlapping ones were the feature genes.

### 2.4 Accuracy assessment

The ROC curve of each differential gene was drawn, and the area under the curve (AUC) was observed and compared. If the AUC is less than 0.7, the accuracy of the gene as a disease diagnosis is low. At the same time, the logical regression model was obtained by comprehensive analysis of each gene, and then the ROC curve of the regression model was drawn to compare the AUC between the single differential gene and the comprehensive model to choose the most accurate model.

### 2.5 Single gene cluster enrichment analysis

GAEA was performed on different groups using the gene set “c2.cp.kegg.symbols.gmt” as a predefined gene set. The importance of the sum pathway and gene sets correlation was explored by normalized enrichment score (NES), nominal *p*-value, and error detection rate (FDR) *q-*values (13). The NES absolute value>1, false discovery rate (FDR) *q*-value<0.25, and nominal *p*-value < 0.05 were considered to be significantly rich. We selected the top seven data sets with a high value of NES.

### 2.6 Single gene set variation analysis

We downloaded the GO and KEGG gene set databases from the Molecular Signatures Database (MSigDB, http://software.broadinstitute.org/gsea/msigdb/index.jsp) on the GSEA website (https://www.gsea-msigdb.org/gsea/index.jsp). “c2.cp.kegg.symbols.gmt” was regarded as the reference gene set. We obtained a matrix file of gene symbols during the above DEGs screening. The matrix files of gene symbols from GO and KEGG databases were processed, and functional pathways were scored according to the absolute enrichment of gene sets in each sample. Additionally, the function and path matrix files mainly containing the GSVA score of the functional pathway corresponding to each sample were got. Next, we used the “limma” package to compare the GSVA scores of OA and normal samples. The criterion for screening differential GSVA score was *p <*0.05 and |log FC| > 0.2 (14).

### 2.7 Evaluation and difference analysis of immune cell infiltration

We evaluated immune cell infiltration in OA using the CIBERSORT algorithm. The immune cell infiltration matrix was obtained with *p* < 0.05.

### 2.8 Correlation analysis of immune cells

To analyze the association between the content of immune cells and the expression of feature genes, we used the “ggplot2” package for correlation analysis and visualization. The relationship between feature genes and differentially infiltrating immune cells was researched by Spearman correlation analysis. Pearson correlation coefficients (r) > 0.6 and p < 0.05 were used to verify the correlation between feature genes and immune infiltrating cells (15).

### 2.9 Drug regulatory network

Drugs targeting feature genes were further revealed using the DGIdb. An online tool called STITCH was used to build a network of interactions between potential drug and feature genes.

### 2.10 Construction of ceRNA network

Three databases were used to predict the target genes of miRNA, including miRanda, miRDB, and TargetScan. The lncRNA-miRNA interaction was predicted by the spongeScan database. Cytoscape was used to construct a ceRNA network.

### 2.11 Culture and extraction of rat chondrocytes

We aseptically obtained the knee cartilage of SD rats aged 3-7 days and then isolated and cultured chondrocytes *in vitro*. The mixture was divided into small pieces and digested with trypsin (Solabio, China) at 37°C for 30min. Then we combined it with collagenase II (1 mg/ml; Gibco) and high glucose DMEM (Gibco, USA) to incubate for 6 hours. And chondrocytes were exclusively collected after centrifugation at the speed of 1500rpm/3min. Gibco (Gibco, USA) and penicillin/streptomycin (Solarbio, China) were used in the DMEM culture medium. Rat chondrocytes were treated with lipopolysaccharide (LPS) to establish an OA cell model. We got approval from the Medical Ethics Committee of the First Affiliated Hospital of Guangxi Medical University (Approval Number: 2023-E115-01).

### 2.12 RNA extraction and qRT-PCR

**Figure 1:**
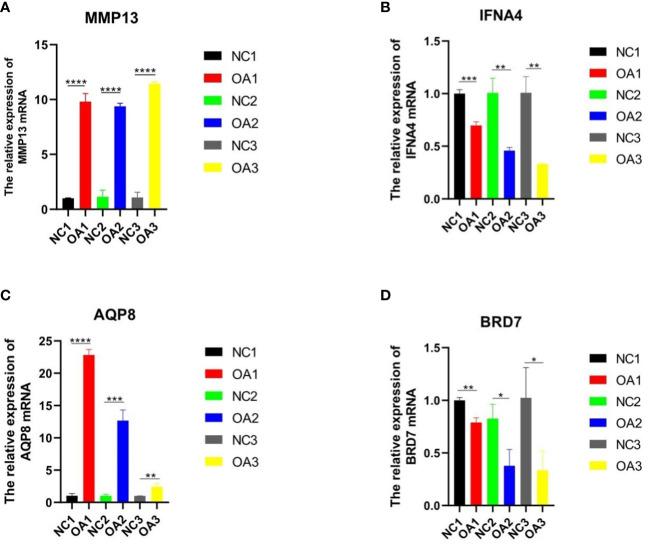
qRT-PCR analysis of three key genes and one inflammatory gene in normal and osteoarthritis SD rat chondrocytes. **(A-D)** The expression of MMP13、IFNA4、AQP8 and BRD7 in the cartilage of normal SD rats and OA rats. “*” means p < 0.05, “**” means p < 0.01, and “***” means p < 0.001.

We extracted total RNA in cells using Hipure Total RNA Minikit (Magen, China), following the manufacturer’s instructions. PrimeScript RT kit and gDNA eraser (Takara, China) were used to reverse-transcribe the extracted RNA into cDNA. Quantitative reverse transcription-polymerase chain reaction (qRT-PCR) was carried out using LightCycler 480 (Roche, Germany) and Fast Start Universal Sybr Green Master Mix (Roche, Germany) under the following circumstances: 10 minutes at 95°C, 15 seconds at 95°C and 1 minute at 60°C. Data from dissolution curves were utilised to confirm the specificity of PCR. We obtained the relative expression levels of genes (MMP13, AQP8, BRD7, IFNA4) by the 2^-ΔΔCt^ method (**Figure 1**). The target mRNA level was standardized to glyceraldehyde-3-phosphate dehydrogenase level (GAPDH). Then they were compared with that of the control group.

### 2.13 Immunohistochemistry

The expression of AQP8, BRD7, and IFNA4 was identified by immunohistochemical staining. The donor agreed on human cartilage samples to apply to this study which was approved by the Medical Ethics Committee of the First Affiliated Hospital of Guangxi Medical University (approval number: 2023-E115-01). After washing human cartilage samples in PBS, cells and sections were exposed to 3% (v/v) hydrogen peroxide at room temperature for 15 minutes to block any endogenous peroxidase activity. Then the cells and areas were blocked for 20 minutes at room temperature with 10% normal goat serum and interacted with the primary antibodies of AQP8 (E-AB-12345), BRD7 (51009-2-AP) and IFNA4 (bs-6304R). The samples were then cultivated with secondary antibodies and biotin-labeled horseradish peroxidase. Before hematoxylin counterstaining, a 3,3’-diaminobenzidine tetrahydrochloride (DAB) kit (ZSGB-BIO, China) was used to observe the situation of antibody binding. Finally the cells were observed and captured with an inverted phase contrast microscope (Olympus BX53) after being dehydrated gradually and sealed with neutral resin.

### 2.14 Statistical analysis

Bioinformatics analysis was carried out in R (x64 4.1.3). The statistical significance of normal distribution variables is calculated by independent sample t test, the significance level is set to p < 0.05, and the legend in the figure indicates the sample size.

## 3 Results

### 3.1 Identify differentially expressed FRGs

**Figure 2:**
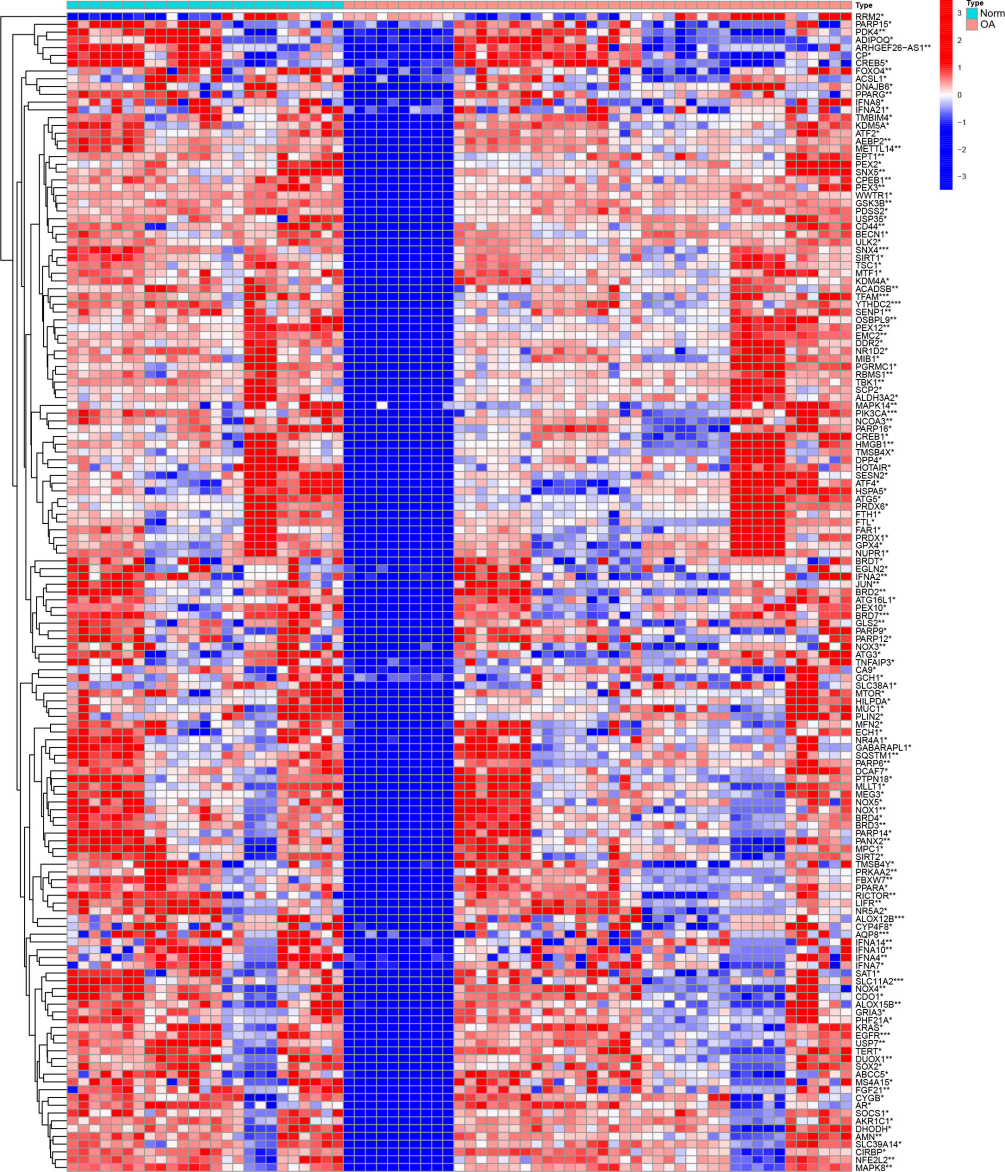
Analysis of DEGs heat map of the dataset. DEGs highly expressed in the samples are marked in red, and DEGs that are low in the samples are indicated in blue. “*” means p < 0.05, “**” means p < 0.01, and “***” means p < 0.001.

**Figure 3:**
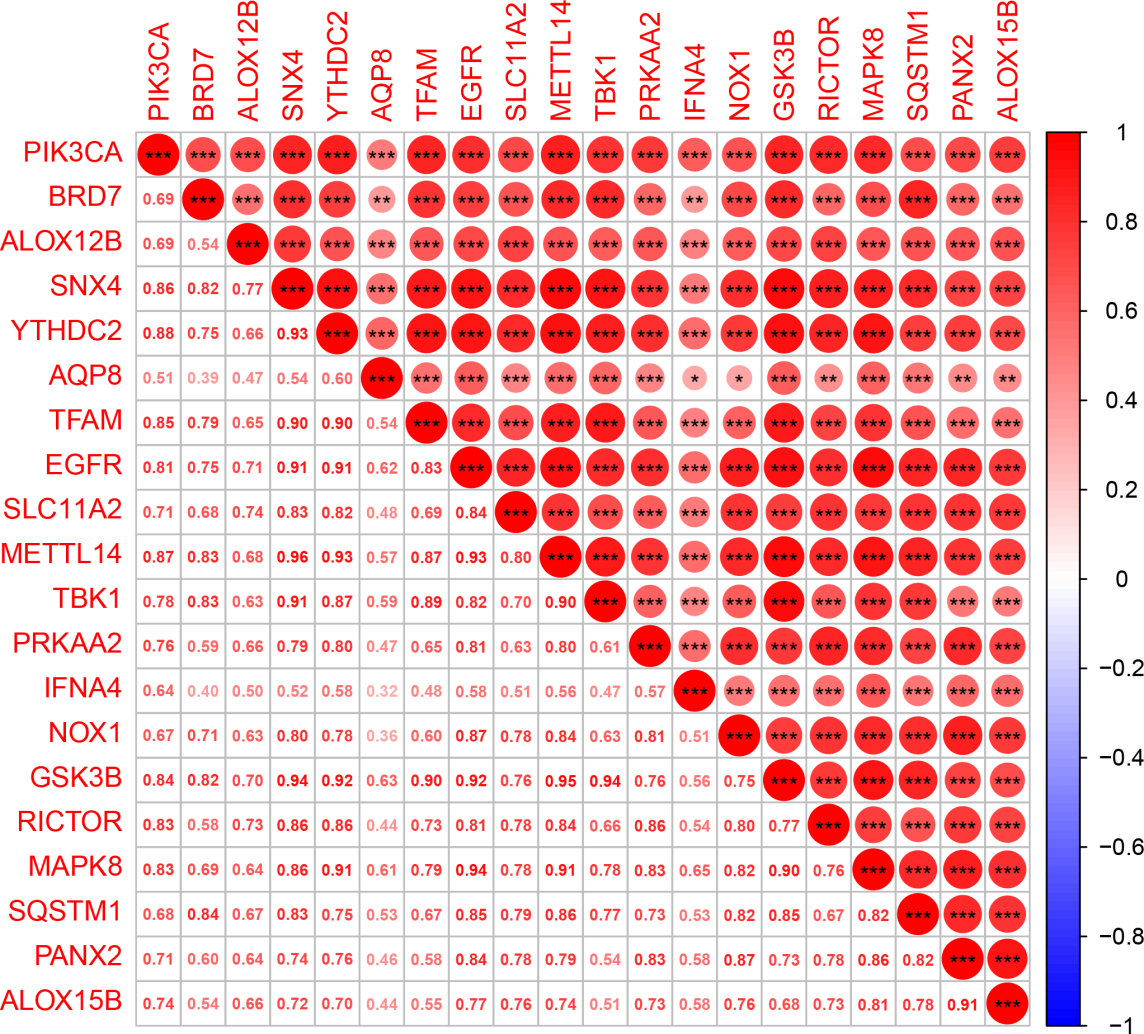
Multi-gene correlation heat map. Red represents gene up-regulation, and the darker the color, the more significant the gene up-regulation. “*” means p < 0.05, “**” means p < 0.01, and “***” means p < 0.001.

The expression matrix of genes was obtained after data correction and standardization, and the ferroptosis-related genes’ expression was then retrieved. 149 DEGs were obtained by differential analysis, including 135 down-regulated genes and 14 up-regulated genes (**Figure 2**). The Spearman correlation analysis of 20 different types of significantly differentially expressed FRGs was displayed in the heat map to investigate these ferroptosis-related genes’ expression correlation. It was discovered that there was a significant positive association among the different genes (**Figure 3**).

### 3.2 GO and KEGG enrichment analyses

**Figure 4:**
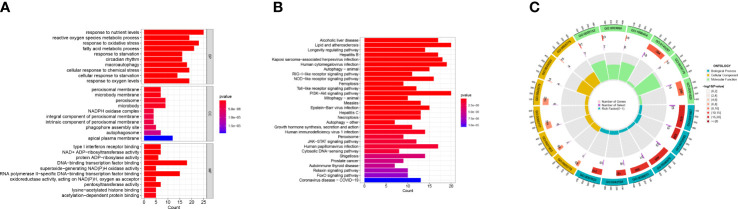
GO enrichment analysis and KEGG pathway analyses. **(A, B)** GO enrichment analysis and KEGG pathway analyses of the 149 DEGs with bar-plot. **(C)** GO enrichment analysis circle can be divided into three types: biological process, cellular component, and molecular function. Different colors represent corresponding types. The innermost circle represents the proportion of genes, and the color represents the color of the second circle. The redder the color, the more significant the enrichment of differential genes in this GO.

We used R software to conduct GO and KEGG enrichment analysis to examine the potential biological activities of these DEGs associated with ferroptosis. The differentially expressed FRGs were primarily involved in the biological process type pathway set and were strongly linked to the response to oxidative stress and fatty acid metabolic process pathway, according to the results of the GO analysis. According to KEGG analysis, most disease pathways, including lipid metabolism, atherosclerosis, and PI3K-Akt signaling pathway, were substantially enriched in differentially expressed FRGs (**Figure 4**).

### 3.3 Screening feature genes

**Figure 5:**
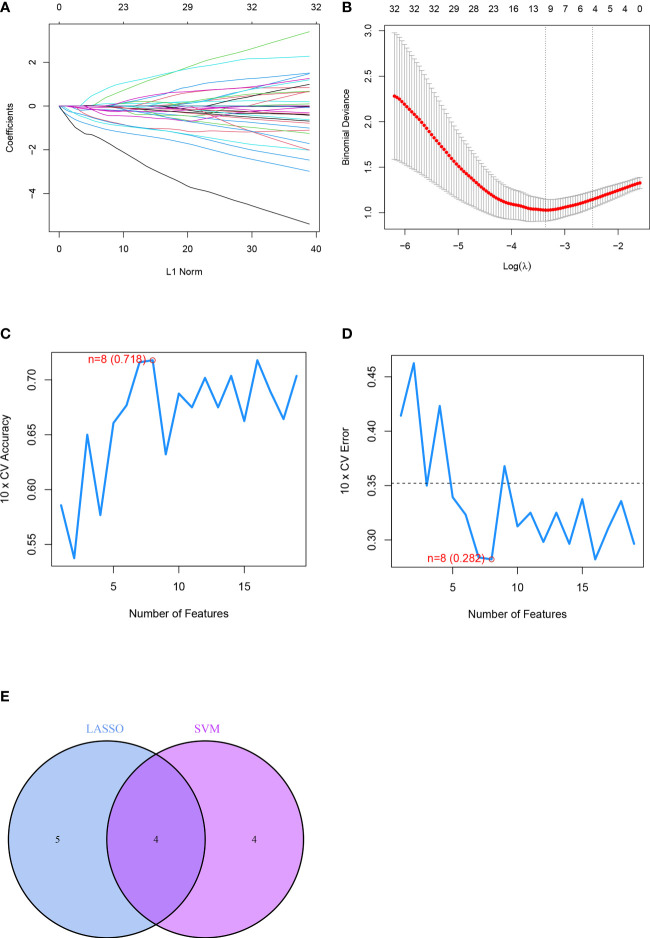
Identification of differential feature genes based on machine learning algorithms included. **(A, B)** Diagram of the optimal number of genes and coefficient spectrum in the LASSO regression model, respectively. **(C, D)** The optimum error rate of the SVM model based on 8 feature genes. **(E)** The Venn diagram showing the overlapping genes in LASSO, SVM modules.

Nine feature genes were screened from the DEGs obtained after the LASSO Cox regression algorithm, which were SLC38A1, ALOX12B, DPP4, TBK1, AQP8, BRD7, IFNA4, IFNA14, and ARHGEF26-AS1 (**Figures 5A, B**). SVM-RFE could find 8 feature genes: AQP8, BRD7, TERT, IFNA4, ABCC5, DCAF7, ARHGEF26-AS1, and ATG16L1 (**Figures 5C, D**). The Veen diagram of feature genes obtained by the two methods was taken to intersect. A total of 4 overlapping genes were obtained: AQP8, BRD7, IFNA4, and ARHGEF26-AS1. These 4 genes were the final feature genes of the disease (**Figure 5E**). In addition, in order to explore the similarity of gene maps between humans and rats, we obtained the protein sequence of characteristic genes between humans and rats in NCBI (https://www.ncbi.nlm.nih.gov/), and compared the sequences. The results show that the gene maps of the two are very similar (**Supplementary Materials 2**, **3**), which suggests that the human diseases in this study can be verified in rats.

### 3.4 Verification of differential genes

**Figure 6:**
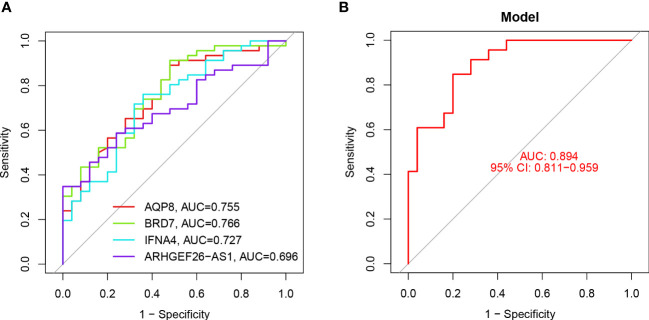
ROC analysis of 4 key DEGs. **(A)** ROC curve analysis of single differentially feature genes. **(B)** ROC curve analysis of the combined differential trait genes.

The feature genes were verified, the ROC curve was drawn, and AUC was compared to judge its diagnostic value. The AUC values of AQP8, BRD7, IFNA4, and ARHGEF26-AS1 were 0.755, 0.766, 0.727, and 0.696, respectively, which had certain diagnostic values, and BRD7 had the highest diagnostic value. At the same time, through the comprehensive analysis of feature genes, the logical regression model was obtained, and the ROC curve was also drawn. We discovered that the complete model’s AUC value was 0.894, which was significantly higher than that of a single gene and suggested that it was more effective at predicting illnesses (**Figure 6**).

### 3.5 qRT-PCR and immunohistochemistry

**Figure 7:**
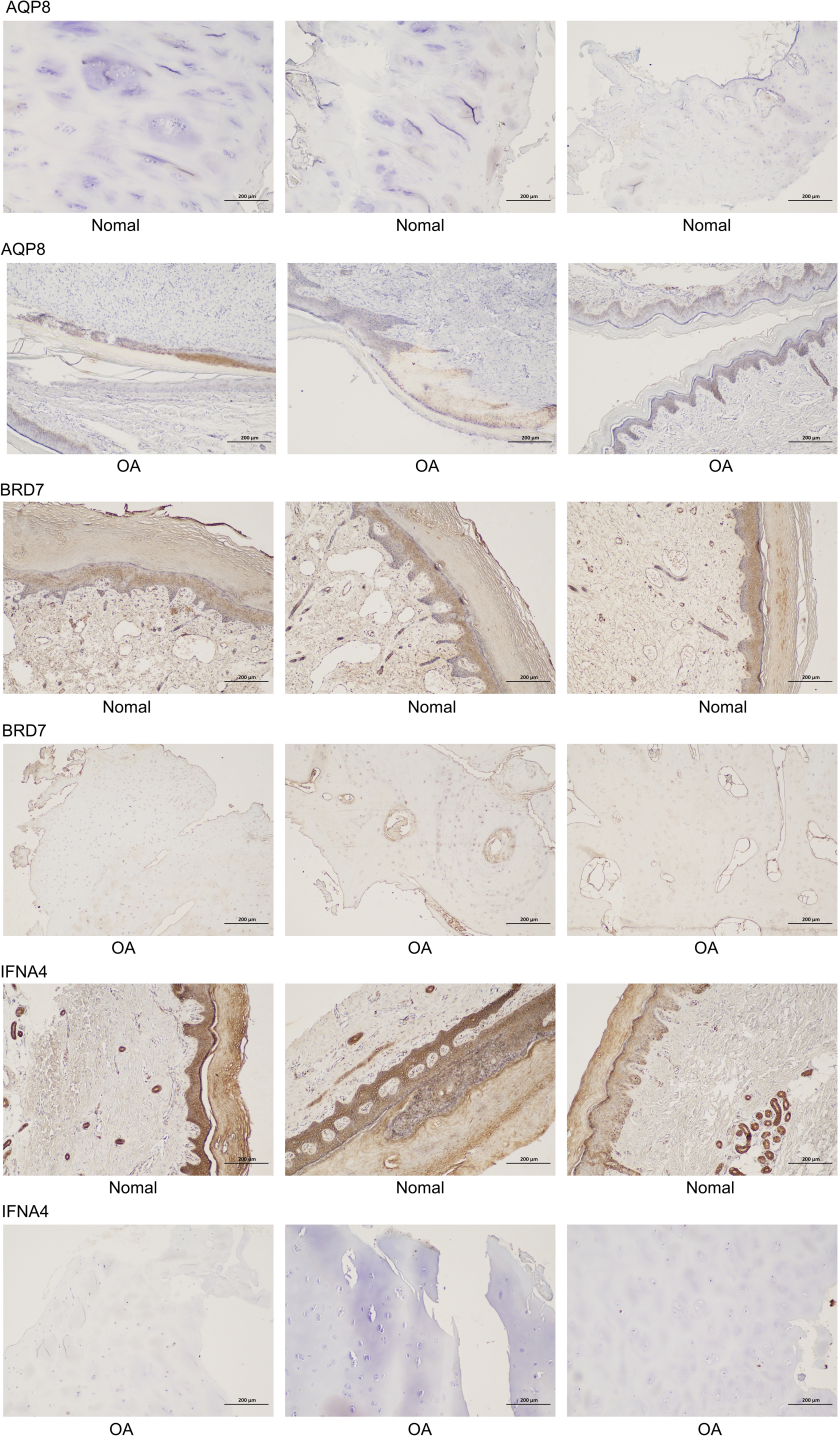
Immunohistochemical study of cartilage in normal people and patients with osteoarthritis.

To verify the expression levels of the key genes mentioned above in OA cells, qRT-PCR analysis was employed to measure the levels of APQ8, BRD7, IFNA4, and in addition MMP13, which is regarded as an inflammatory-related gene marker (**Table 1** shows the primer sequence). Compared with normal rat chondrocytes, the expressions of APQ8 and MMP-13 in lipopolysaccharide-treated rat chondrocytes increased substantially, while that of BRD7 and IFN-A4 had an obvious decrease (P < 0.05), which meets the results in above analysis (**Figure 2**). Besides, we collected 3 normal and 3 osteoarthritis cartilage samples from the First Affiliated Hospital of Guangxi Medical University. Then, we detected and contrasted the expression of AQP8, BRD7 and IFNA4 by histochemical staining in two groups. The results demonstrated that the positive rate of AQP8 in the OA group was higher than normal (**Figure 7**). In contrast, strong positive staining of BRD7 and IFNA4 could be observed in the superficial and middle cartilage of the normal group, further confirming our research results.

**Table 1:** Primer sequences used in qRT-PCR experiments.

Gene name	Forward primer	Reverse primer
GAPDH	AGTGCCAGCCTCGTCTCATA	GGTAACCAGGCGTCCGATAC
MMP-13	CGCCTCGAGATGCATCCAGGGGTCCTGGCT	CCCGTCGACCCTACCCCGCACTTCTGGAAGTATTA
APQ8	TGTGTGTATGGGTGCCGTCAATG’	GCCATCACAGCAGGTCCAAAGG
BRD7	AAGAAGCTGTTGCACTCAGGGATG	AGCCACTGTCCTCTCCACCTTG
IFNA4	CTGCTGGCTGTGCGGGAATAC	CCACACTTCTGCTCTGACCACTTC

### 3.6 Feature genes are closely associated with various pathways related to ferroptosis in OA

**Figure 8:**
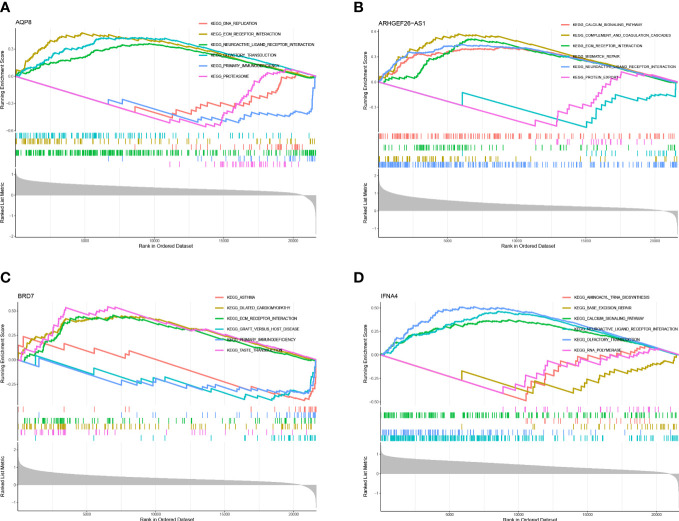
GSEA of differential feature genes. **(A)** GSEA of AQP8. **(B)** GSEA of ARHGEF26-AS1. **(C)** GSEA of BRD7. **(D)** GSEA of IFNA4.

**Figure 9:**
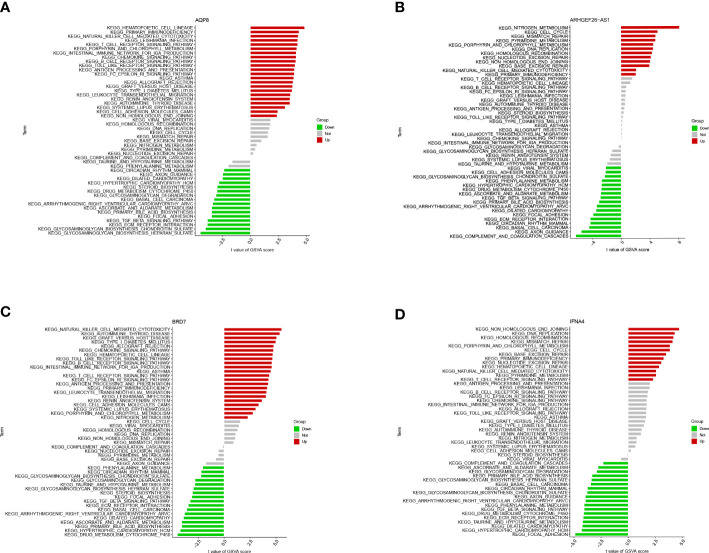
GSVA analysis of the 4 differential feature genes. **(A)** GSVA analysis of AQP8. **(B)** GSVA analysis of ARHGEF26-AS1. **(C)** GSVA analysis of BRD7. **(D)** GSVA analysis of IFNA4.

Single gene GSEA analysis was conducted to investigate further feature genes’ possible role in differentiating between sick and normal samples. The figure reflected the characteristics of each feature gene regulatory pathway. We discovered after a thorough investigation that these genes were abundant in the cell cycle, immunological response, protein synthesis, and metabolic pathways, all of which were linked to ferroptosis in OA. We then examined the varied activation pathways between groups with high and low expression levels of each marker gene binding to GSVA. The findings demonstrated that the decreased level of BRD7 transcription in the illness activated the main immunodeficiency pathway, and the overexpression of it might induce the ferroptosis of OA by activating the extracellular matrix (ECM) receptor interaction pathway and inhibiting the primary immunodeficiency pathway. The upregulation of AQP8 activated the ECM receptor interaction pathway and inhibited the primary immunodeficiency pathway. In contrast to IFNA4, whose low expression was associated with the DNA replication and protein synthesis pathways, while the high expression of this gene activated the calcium signal transduction pathway and inhibited the DNA replication and protein synthesis pathway, ARHGEF26-AS1 high expression group activated calcium signal transduction pathway, complement pathway, and ECM receptor interaction pathway. The high expression group of these genes enriched a range of pathogenesis-related pathways of ferroptosis in OA (**Figures 8**, **9**).

### 3.7 Correlation and difference analysis of immune infiltration

**Figure 10:**
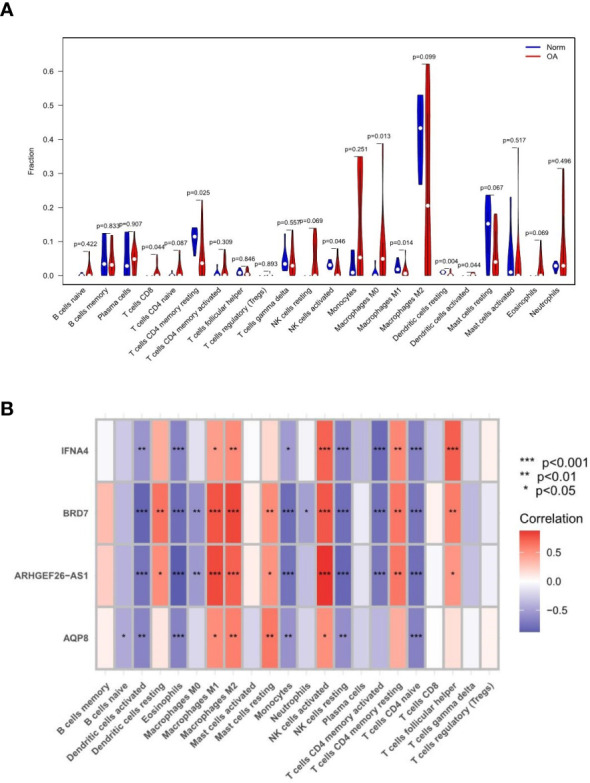
Results of immune infiltration by CIBERSORT. **(A)** Split violin presented the different immune infiltration of 22 immune cells. **(B)** Bar-plot showed 22 kinds of immune cell composition in control and experimental groups. “*” means p < 0.05, “**” means p < 0.01, and “***” means p < 0.001.

Monocytes and macrophages comprised most of the invading cells, particularly in OA tissues. **Figure 10A** depicts the variance in penetration between the two groups. Compared with the healthy control group, 7 types of immune cells, including CD8(+) T cells, CD4(+) T cells, natural killer (NK) cells activated, M0 macrophages, M1 macrophages, M2 macrophages, and dendritic cells activated, had differential infiltration in OA patients and were up-regulated in OA tissues. **Figure 10B** depicts the relationship between feature gene expression and differently invading immune cells. It was observed that BRD7, AQP8, IFNA4, and ARHGEF26-AS1 had positive correlations with macrophages, activated NK cells, and memory T cells. However, a strong negative correlation existed between BRD7, AQP8, IFNA4, and ARHGEF26-AS1 and activated dendritic cells, eosinophils, monocytes, resting NK cells, memory-activated CD4(+) T cells, and naive CD4(+) T cells. The expression of most feature genes shown in the heat map is also linked with the expression of other immune cells weakly to moderately positively and negatively (16).

### 3.8 Prediction of targeted drugs with feature genes

**Figure 11:**
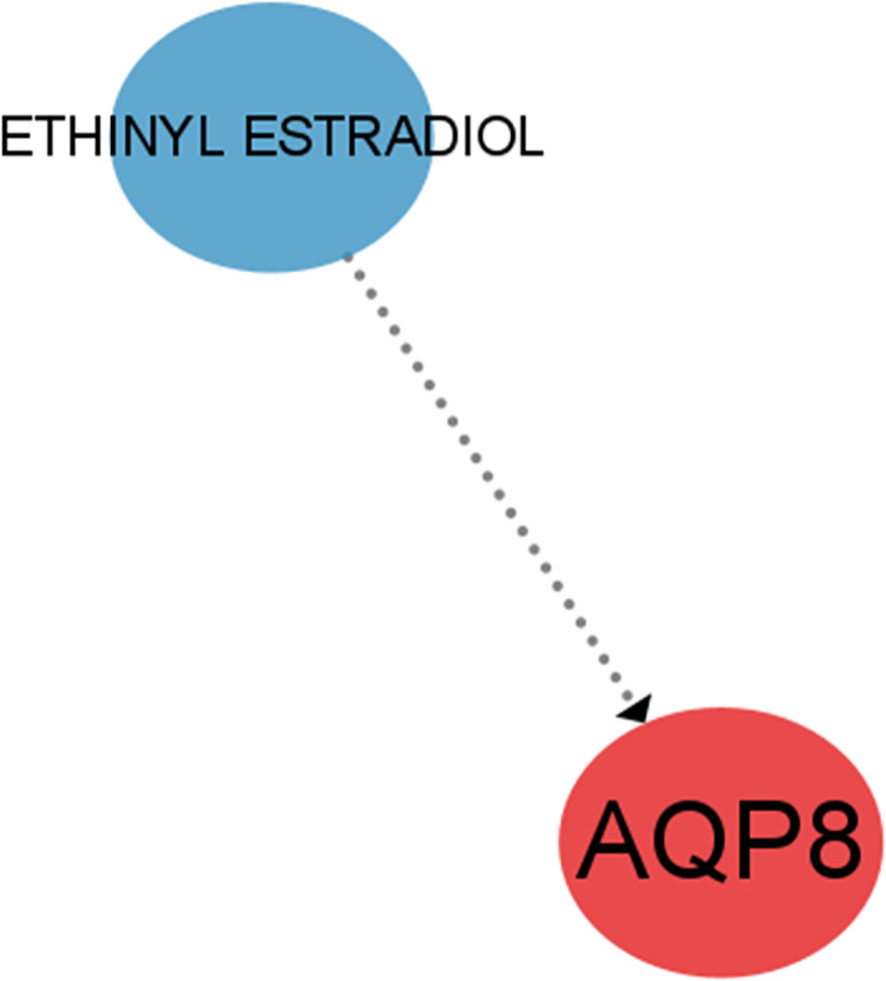
Targeted drugs based on the DGIdb database for predicting differential feature genes.

Through the DGIdb database, we further identified the potential pharmacological targets and used CIBERSORT to visualize them. A possible targeting drug ETHINYL ESTRADIOL for AQP8, was found (**Figure 11**).

### 3.9 Marker gene-based CeRNA network

**Figure 12:**
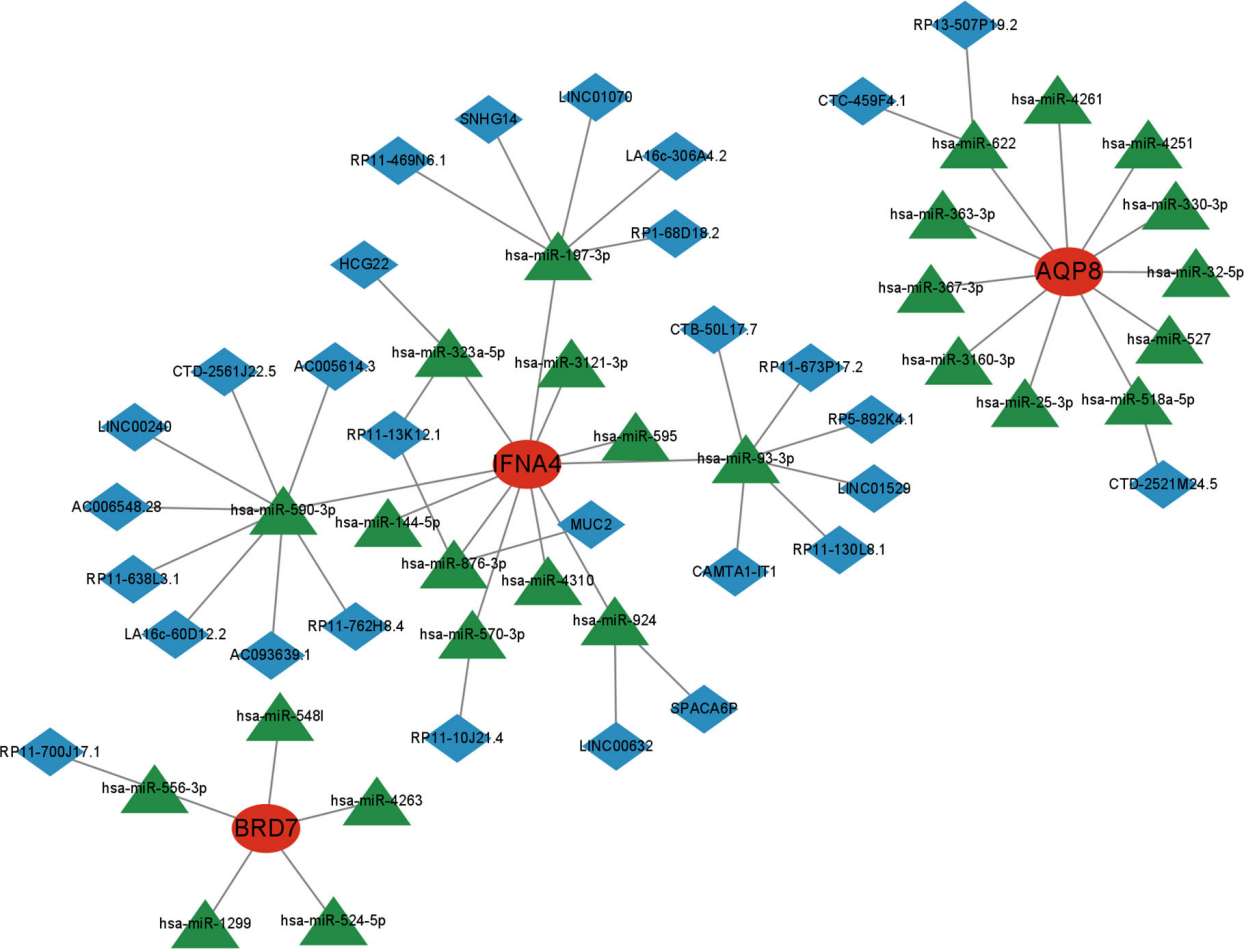
A ceRNA networks of AQP8, BRD7, IFNA4, and the potential RNA regulatory pathways.

Next, we utilized the starBase and miRanda databases to build a ceRNA network based on four feature genes. The network comprised 59 nodes (3 marker genes, 27 miRNAs, and 29 lncRNA). We discovered that the combination of hsa-miR-197-3p, hsa-miR-590-3p, hsa-miR-93-3p, hsa-miR-323a-5p, hsa-miR-876-3p, hsa-miR-570-3p, and hsa-miR-924 may competitively regulate IFNA4. We discovered that a lncRNA could control the expression of BRD7 by antagonistic binding to hsa-miR-556-3p. In AQP8’s ceRNA network, 2 and 1 lncRNA can respectively bind to hsa-miR-622 and hsa-miR-518a-5p to regulate the gene (**Figure 12**).

## 4 Discussion

Ferroptosis is a new horizon for current research to explore new biomarkers and therapeutic targets, but the research in OA is still incomplete. In this study, we found 4 feature genes related to ferroptosis in OA; they inspired our understanding of the relationship between ferroptosis and the OA mechanism. Also, we looked into new treatment options using immune infiltration analysis, which could help find new OA biomarkers and therapeutic targets.

Although OA is an illness without a clear explanation of its occurrence and progression, several involved research has shown that the pathogenesis of OA is closely related to changes in some specific signaling pathways, abnormal mechanical load, and ageing (17). In this paper, 149 FRGs were significantly expressed in OA, and the GO functional enrichment analysis indicated that many of them were enriched in the biological process, including oxidative stress and fatty acid metabolism pathways. KEGG pathway enrichment analysis revealed that differentially expressed FRGs were mainly enriched in lipid and atherosclerosis, necroptosis, autophagy and PI3K/Akt pathway.

Several recent studies have revealed that the PI3K/Akt signal transduction pathway has a role in several conditions that affect the bones, including osteoporosis, OA, and osteosarcoma. Moreover, it significantly impacts the proliferation, differentiation, and apoptosis of osteoclasts and osteoblasts (18). Sun et al. found that the PI3K and Akt content decreased in OA (18), which may lead to a reduction in the PI3K/Akt pathway activity. Then reduces the activity of its downstream target mTOR, a key autophagy inhibitor, resulting in abnormal activation of cellular autophagy in OA and synergistic activation of massive chondrocyte apoptosis in the joint (19). At the same time, mitochondrial autophagy, a form of selective autophagy, engulfs large amounts of ferritin, leading to excessive Fe^2+^ release, increasing the intracellular pool of unstable iron and thus leading to chondrocyte ferroptosis (4). Several papers have demonstrated that the PI3K/Akt has a critical effect on the occurrence of OA in terms of maintaining cartilage homeostasis, regulating the differentiation and proliferation of osteoblasts in subchondral bone, and regulating synovial inflammation (18). Improving the activity of this pathway in OA may delay the development of the disease and enhance the expression of downstream genes and signal transduction to inhibit the ferroptosis of synovial fibroblasts (SFs) (20), which is also important for the study of ferroptosis. In this way, a novel potential therapy for OA is provided.

In addition, Basu et al. found that human OA cartilage has higher lipid peroxidation levels than normal cartilage (21); this may be due to reduced glutathione (GSH) levels or becoming more oxidized under conditions such as excessive mechanical loading of the joints and ageing, resulting in reduced resistance to oxidative stress. The abnormal reduction in intracellular GSH content eventually inactivates glutathione peroxidase (GPX4) and leads to the accumulation of lipid peroxidation, which induces cellular ferroptosis after reaching a certain level (22), which may shed some light on how differential FRGs may participate in ferroptosis-related OA.

The NOD-like receptors (NLR) family, widely present in the cytoplasm of human cells, can activate the NF-κB signaling pathway after recognizing the corresponding ligands, next activates caspase-1 to produce the pro-inflammatory factors like IL-1β and IL-18, thereby initiating the development of intrinsic and acquired immunity (23). Cytoplasmic DNA produced by cartilage damage in OA can activate Caspase-1, which binds to the inflammasomes generated by the activation of NLRP3, leading to a large generation of reactive oxygen species (ROS), and excessive accumulation can induce ferroptosis. The activation of NLRP3 inflammatory vesicles in the NOD-like signaling pathway and the production of inflammatory factors may positively influence the developing ferroptosis in OA. All of the pathways mentioned include the activation of the NF-κB pathway, suggesting its involvement in the process of ferroptosis in OA, but the exact mechanism needs to be further explored.

Through two machine-learning intersections, we identified 4 feature genes (BRD7, AQP8, IFNA4, and ARHGEF26-AS1), but the extent of their involvement in the development of ferroptosis in OA remains to be further investigated. We plotted ROC curves for each feature gene to validate internally, and all 4 genes’ AUC values were larger than or almost equal to 0.7, indicating that they have certain diagnostic significance. It suggests that these genes may be biological markers of ferroptosis in OA.

GSEA and GSVA analysis of the 4 feature genes revealed that they are enriched in the cell cycle, immune response, protein synthesis, and metabolic pathways. Several studies have shown that an important characteristic of OA is immune cell infiltration. We performed immune cell-related analyses to learn what role these immune cells play in OA. The content of each cell in the experimental and control groups of each data set was extracted and analyzed for differences. It has been shown that synovitis in OA is dominated by macrophage infiltration, reflecting the activation of the innate immune response (24). The predominance of activated NK cells, M0 macrophages, and dendritic cells activated in our results are consistent with this feature. Inflammatory transcription factors induced by autoantibody or protein are enhanced by ferroptosis-catalyzed oxidation, producing more matrix, cytokine/chemokine production and immune cells (25). Immune infiltration analysis outcomes also indicated a positive relationship between OA and CD8(+) T cells, activated NK cells, M0 macrophages, and dendritic cells activated. It has been shown that CD8 T cells have a biphasic role in OA, on the one hand controlling CD8(+) T cells dissolving T cells through the PI3K/Akt/mTORC1/HIF1 pathway; on the other hand, CD8(+) T cells in animal models kill DCs and abnormal T cells in a perforin-dependent manner (26), thus promoting inflammation. In addition, some studies have revealed that ferroptosis can coordinate CD8(+) T cells and thus enhance cellular immunity to achieve anti-tumor effects (27). Therefore, we hypothesize that increased CD8(+) T cells involved in ferroptosis may take part in the pathogenesis of OA. Besides, immune infiltration analysis suggests that CD4(+) T cells memory resting, activated NK cells, M1 macrophages, and dendritic cells resting cells are reduced, which may lead to decreased ferroptosis cell clearance in the osteoarthritic environment, ultimately exacerbating the development of OA.

One of the 4 feature genes, BRD7 is an oncogene widely expressed in various human tissues and has been extensively studied as a nasopharyngeal carcinoma-associated gene (28). It can interact with other transcription factors involved in transcriptional regulation, such as competing with p110 for interaction with p85α to attenuate PI3K activity (29). It links to the previously mentioned PI3K/Akt signaling pathway and explains the downregulation of PI3K activity in OA. Of interest, Zhang et al. found that BRD7 overexpression induced classical ferroptosis, BRD7 knockdown resisted ferroptosis events and that ferroptosis mediated by high expression of BRD7 may be related to its binding to the N-terminal inverse activation domain of p53 thereby promoting translocation expression of the p53 gene in mitochondria (30). Meanwhile, it has been demonstrated that low expression of BRD7 in the disease activates the primary immunodeficiency pathway. Thus, we speculate that overexpression of BRD7 may induce ferroptosis in OA by inhibiting the primary immunodeficiency pathway. However, further investigations need to be accomplished on the precise mechanism.AQP8, located on chromosome 16, region p12 (31), encodes a transmembrane water channel protein and hydrogen peroxide transporter protein implicated in various cell deaths. A study on pancreatitis suggested that the RIPK1/NF-κB/AQP8 axis may inhibit acinar cell necrosis (32). Over-expressed AQP8 in colorectal cancer (CRC) cells can inhibit the PI3K/Akt signaling transduction (31), which may have a similar effect in OA, and in concert with BRD7, leads to downregulation of PI3K activity in OA. We visualized the drug-gene interactions through cytospace to construct a drug regulatory network and eventually identified a possible drug that regulates AQP8, ethinyl estradiol, an estrogen component of almost all oral contraceptives (OCs) (33). Given the high incidence of OA and the significant changes in sex hormones in women after menopause around the age of 50, it has been found that the lack of estrogen decreases the expression of Akt (34), causing increased chondrocytes apoptosis and articular cartilage degeneration, thereby promoting the development of OA. Our search for this drug may then offer a possibility for the treatment of OA through estrogen regulation of AQP8 expression.

We also mapped the ceRNA network with the feature genes at its center. lncRNAs can compete with mRNAs to bind to miRNAs, thereby regulating the expression of 4 feature genes (14). Wang et al. explored targeting lncRNAs for therapy by identifying OA signature lncRNAs (35), suggesting that these miRNAs that bind directly to mRNAs and lncRNAs that regulate mRNA expression indirectly may provide new possible targets for the prevention or treatment in OA.

There are a few non-negligible drawbacks in this study. Above all, the data we chose were only retrieved from the GEO database, which is an insufficient sample size, and IFNA4 also lacks relevant external verification at present. The next step requires validation of each gene and the combined model constructed from the 4 genes in a larger dataset. In addition, although the biological functions of 4 feature genes were validated in cancer or other diseases, it is noteworthy that these disease models have different biological characteristics from OA, and their specific roles in OA need further investigation. What’s more, there is a lack of valid experimental evidence to confirm the therapeutic effect between the drugs found and OA. Finally, more research is needed regarding the specific association of lncRNAs and miRNAs with signature genes.

## Conclusion

In summary, this research selected 4 differentially feature genes that can be potential biomarkers of ferroptosis in OA, including AQP8, BRD7, IFNA4, and ARHGEF26-AS1. The possible mechanism of four key genes in osteoarthritis was explored through analysis. The recently identified lncRNAs in the ceRNA network results and the newly discovered AQP8-targeting medication ETHINYL ESTRADIOL may provide fresh concepts for targeted therapy in OA.

## Data availability statement

The original contributions presented in the study are included in the article/**Supplementary Material**. Further inquiries can be directed to the corresponding author.

## Ethics statement

The studies involving human participants were reviewed and approved by First Affiliated Hospital of Guangxi Medical University Ethical Review Committee. The patients/participants provided their written informed consent to participate in this study. The animal study was reviewed and approved by First Affiliated Hospital of Guangxi Medical University Ethical Review Committee.

## Author contributions

JY designed the study and revised the manuscript. YQ, LL and MX conducted data analysis and experiments. XH and JM wrote a draft manuscript. All authors contributed to the article and approved the submitted version.

## Supplementary material

Supplementary material 1 Relative Logarithmic Representation of Merged Datasets.

Supplementary material 2 and 3 Comparison of protein sequences between humans and rats.
