# Supplementary figures and images for: Machine learning identifies ferroptosis-related genes as potential diagnostic biomarkers for osteoarthritis
Source: Front Endocrinol (Lausanne). 2023 Jun 12;14:1198763. doi: 10.3389/fendo.2023.1198763 (PMC10292652; doi:10.3389/fendo.2023.1198763)

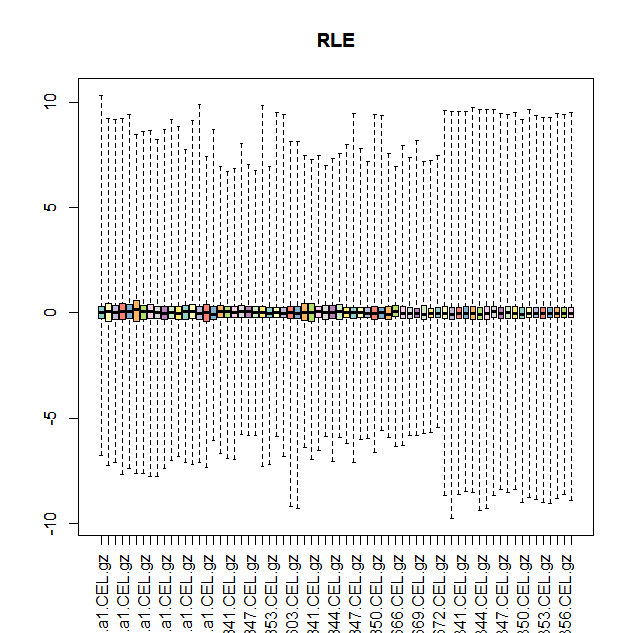

Supplement: Supplementary material 1 — Relative Logarithmic Representation of Merged Datasets. [file Image_1.png]

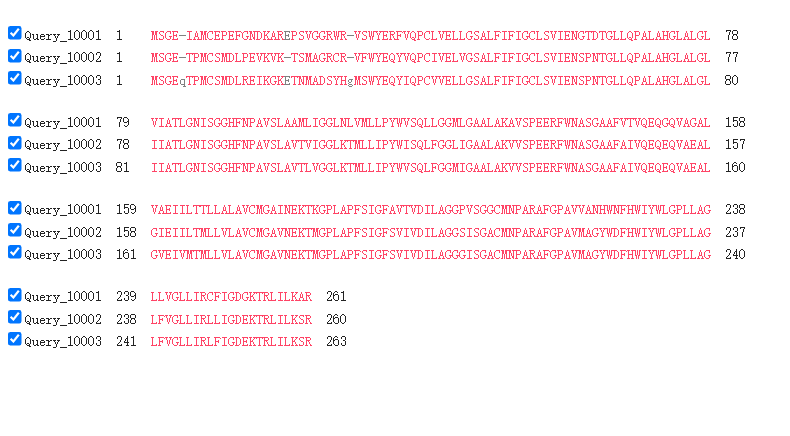

Supplement: Supplementary material 2 and 3 — Comparison of protein sequences between humans and rats. [file Image_2.jpeg]

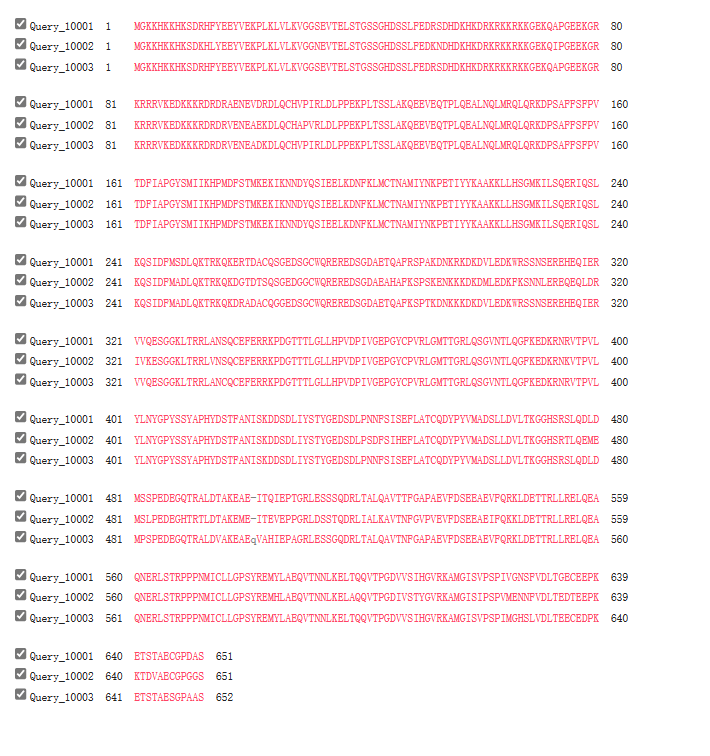

Supplement: Supplementary file 3 [file Image_3.jpeg]
